# Association between Skeletal Muscle Mass-to-Visceral Fat Ratio and Dietary and Cardiometabolic Health Risk Factors among Korean Women with Obesity

**DOI:** 10.3390/nu15071574

**Published:** 2023-03-24

**Authors:** Heeju Lim, Kumhee Son, Hyunjung Lim

**Affiliations:** 1 Department of Medical Nutrition, Graduate School of East-West Medical Science, Kyung Hee University, Yongin-si 17104, Gyeonggi-do, Republic of Korea; 2 Research Institute of Medical Nutrition, Kyung Hee University, Seoul 02447, Republic of Korea

**Keywords:** sarcopenic obesity, skeletal muscle mass-to-visceral fat area ratio, cardiometabolic risk factors, dietary factors

## Abstract

Sarcopenic obesity (SO) is more associated with cardiovascular diseases than sarcopenia or obesity alone. This study aimed to assess the association between the skeletal muscle mass-to-visceral fat area ratio (SVR) and dietary and cardiometabolic health risk factors in obese women. Fifty-nine women aged 19–65 years with BMI values of ≥25 kg/m^2^ and <32 kg/m^2^ were included. The SVR was inversely correlated with blood lipids (total cholesterol, β = −0.369, *p* = 0.022; low-density lipoprotein cholesterol, β = −0.326, *p* = 0.049) and hs-CRP (β = −0.305, *p* = 0.043). Among the dietary factors, fatty acid intake (saturated fatty acids (SFA), β = −0.287, *p* = 0.044; monounsaturated fatty acids (MUFA), β = −0.282, *p* = 0.048; polyunsaturated fatty acids (PUFA), β = −0.301, *p* = 0.035) was inversely correlated with the SVR. Conversely, vitamin B_6_ and B_12_ intake (vitamin B_6_, β = 0.338, *p* = 0.012; vitamin B_12_, β = 0.281, *p* = 0.024) showed positive associations with the SVR. Individuals with a lower SVR were more likely to have SO and higher blood lipids and inflammatory marker levels. Regarding dietary factors, the SVR increased with vitamin B_6_ and B_12_ intake, which was less likely to occur in individuals with SO.

## 1. Introduction

Sarcopenic obesity (SO) is a combination of obesity and sarcopenia. It is characterized by decreased muscle mass and increased body fat with aging. The amount of appendicular lean mass decreases with age, which reduces the usual walking pace and total physical activity. As the appendicular lean mass and walking pace decrease, physical activity and basal metabolic rate also decrease, causing an increase in visceral fat and body mass. This process leads to SO [1,2]. In addition, obesity causes fat accumulation in the muscles, which may promote insulin resistance, oxidative stress, and pro-inflammatory responses in overweight and obese populations, leading to sarcopenia [3]. Factors such as body fat, grip strength, muscle mass, and gait speed, which affect the occurrence of SO, may be partially influenced by genetics as well as aging [4]. Hence, SO can occur not only in the elderly but also in people with related genes, regardless of age. The prevalence of SO is higher in women in Korea [5]. According to a study of elderly Koreans, the prevalence of SO was 6.1% in men and 7.3% in women [6]. Another study of Koreans aged over 20 years showed a prevalence of SO of 7.6% in men and 9.1% in women [7]. 

SO has attracted significant attention because those with SO have a higher risk of diseases such as metabolic syndrome and cardiovascular disease than those with either sarcopenia or obesity alone [8]. The factors affecting SO are visceral fat and skeletal muscle mass, which are important determinants of cardiometabolic risk [9,10]. However, there is a lack of standard criteria for diagnosing SO [11]. Moreover, the diagnostic criteria used in several studies are based on the independent diagnosis of sarcopenia and obesity without the simultaneous consideration of visceral fat and muscle mass. The most commonly used SO index is calculated as: appendicular skeletal muscle mass (ASM)/height^2^. However, since it does not include visceral fat, it is more likely to underestimate sarcopenia in individuals with obesity [12]. Few studies related to nutrition consider visceral fat and muscle mass together. Therefore, to analyze the relationship between SO and cardiometabolic risk factors in adult women with obesity we used the appendicular skeletal muscle mass-to-visceral fat area ratio (SVR), which can predict SO by considering both muscle and visceral fat mass [13].

Nutritional measures are also being considered in preventing SO. Although there is a relationship between other indexes of SO and nutrition [1], there are few studies analyzing the relationship between the SVR and dietary factors. Therefore, the current study aimed to investigate the association between the SVR and dietary and cardiometabolic risk factors in adult Korean women with obesity based on anthropometric and biochemical indices, blood pressure measurements, and questionnaire data. 

## 2. Materials and Methods

### 2.1. Study Subjects

This study analyzed the baseline cross-sectional data of a study conducted from November 2020 to June 2021 at the Kyung Hee Medical Center. It was approved by the institutional review committee of Kyung Hee Medical Center (IRB approval No. KHUH2020-01-051). Subjects were recruited through posters at the Kyung Hee Medical Center and subway advertisements. In total, 53 Korean women aged 19–65 years with body mass index (BMI) values ≥25 kg/m^2^ and <32 kg/m^2^ who filled out the consent form were included in the analysis. Individuals who took drugs affecting weight, psychiatric drugs such as those for depression, beta blockers, diuretics, contraceptives, steroids, and female hormones were excluded. Additionally, people who consumed functional foods, supplements for obesity, who could not exercise due to musculoskeletal disorders, and who had chronic diseases affecting blood indicators were not included in this study. 

### 2.2. Socio-Demographic and Health-Related Characteristics of the Subjects

Data on sociodemographic factors such as age, educational level, household members, and menopausal status were investigated by an experienced assistant. Information on health-related characteristics such as medical history, alcohol consumption, smoking status, protein supplementation, and physical activity was also obtained. Alcohol consumption was categorized as non-drinker, former drinker, and current drinker. Smoking status was classified similarly. Physical activity was investigated using the Korean version of the Global Physical Activity Questionnaire (GPAQ) [14]. The amount of physical activity during the week was classified as activity at work, travel to and from places, recreational activity, and total physical activity. Each amount of physical activity was calculated as the metabolic equivalent (MET) value. The MET value was calculated according to the GPAQ analysis guidelines [15]. The sedentary time in minutes was documented.

### 2.3. Anthropometric and Blood Pressure Measurements

The percentage of body fat (%), body fat mass (kg), and lean body mass (kg) were measured using DXA (Lunar iDXA, GE Healthcare, Chicago, IL, USA). Computed tomography (CT) (Ingenuity CT, Siemens Healthineers, Erlangen, Germany) was used to measure the visceral fat area (VFA) (cm^2^) and subcutaneous fat area (cm^2^). The CT measured the fat area between the 4th and 5th lumbar vertebrae ranging from −110 to −100 Hounsfield Units (HU). Height (cm) was measured using a stadiometer (DS-103, Dong Sahn Jenix, Seoul, Korea), and body weight (kg) and BMI (kg/m^2^) were measured using bioelectrical impedance analysis (BIA; Inbody 720 body composition analyzer, Inbody Co., Seoul, Korea). Waist circumference (WC) was measured between the lowest rib and iliac crest, and hip circumference (HC) was measured at the top of the hip, both with a tape measure to the nearest 0.1 cm while the subjects were wearing light clothing. Waist–hip ratio (WHR) was calculated as: WC (cm)/HC (cm). Systolic blood pressure (SBP) and diastolic blood pressure (DBP) were measured on the right arm of the subjects, after maintaining a stable condition for at least 10 min, using an automatic sphygmomanometer (BPBIO-320, Inbody Co., Seoul, Korea).

### 2.4. Biochemical Measurements 

Before blood sampling, all subjects fasted for at least 8 h. To evaluate leptin (ng/mL), and adiponectin (μg/mL) concentrations, samples were collected and stored in a serum separation tube (with 5 mL CAT Serum Sep Clot Activator). After coagulation, the samples were centrifuged (UNION 32R PLUS, Hanil Science Co., Ltd., Daejeon, Korea) at 3000 rpm for 10 min to separate leptin and adiponectin. After separation, leptin and adiponectin were stored in a freezer at −20 to 0 °C. After freezing, leptin and adiponectin were analyzed at the GC Medical Foundation (Gyeonggi-do, Korea). The adiponectin-to-leptin ratio was obtained by dividing adiponectin by leptin. The Homeostatic Model Assessment of Insulin Resistance (HOMA-IR) was used to measure insulin resistance and calculated using the following formula: fasting insulin (uIU/mL) × fasting glucose (mg/dL)/405. Fasting blood sugar (FBS) (mg/dL) was analyzed by an enzymatic colorimetric glucose oxidase assay (ADAMS glucose GA-1171, Arkray, Kyoto, Japan)**.** Venous blood was collected in a separate tube for serum insulin (uIU/mL) measurement by radioimmunoassay (Gamma-10, Shinjin medics, Seoul, Korea). Serum lipid levels of triglyceride (TG) (mg/dL), total cholesterol (mg/dL), HDL-cholesterol (HDL-C) (mg/dL), and LDL-cholesterol (LDL-C) (mg/dL) were determined using an enzymatic assay (AU5800, Beckman Coulter, Brea, CA, USA). High-sensitivity C-reactive protein (hs-CRP) (mg/dL) was analyzed using a nephelometry assay (BN II nephelometer, Dade Behring, Deerfield, IL, USA).

### 2.5. Sarcopenic Obesity Diagnostics and Subject Classification

SO was diagnosed using the ASM-to-VFA ratio (SVR) (g/cm^2^). The ASM was obtained by adding the lean soft tissues of both arms and both legs measured via DXA. The SVR was calculated as ASM (g) divided by VFA (cm^2^). The participants were divided into low SVR (<141.21 g/cm^2^) and high SVR (SVR ≥ 141.21 g/cm^2^) groups, based on the median value. The lower the SVR, the higher the VFA and lower the ASM, which suggested SO. To measure the accuracy of SVR, the ASM-to-BMI ratio (SBR), ASM-to-total fat mass ratio (SFR), and ASM-to-WC ratio (SWR) were used. The SBR was calculated as the ASM divided by the BMI, and SFR was obtained by dividing the ASM by the total fat mass. Additionally, the SWR was obtained by dividing the ASM by the waist circumference [16].

### 2.6. Dietary Assessment 

The participants’ 3-day food record was used to assess nutrient and daily food intake. It was recorded for 3 days a week (2 weekdays and 1 weekend day). An experienced dietitian checked the 3-day food record against food models written by the participants in advance. The daily nutrient intake per day was calculated using CAN-pro 5.0 (CAN-pro 5.0; Computer Aided Nutritional analysis program, Korean Nutrition Society). Data were analyzed based on the average intake over 3 days.

Eating behavior was assessed using the Dutch Eating Behavior Questionnaire (DEBQ) [17]. All subjects answered 33 questions. The restrained eating subscale (questions 1–10) assessed the effort to control food intake due to weight concerns. The emotional eating subscale (questions 11–23) assessed eating behavior due to various emotions. The external eating subscale (questions 24–33) assessed eating behavior in response to food-related stimuli. 

### 2.7. Statistical Analysis

All statistical analyses were performed using SAS version 9.4 (SAS Institute, Cary, NC, USA), and a *p*-value of <0.05 was considered statistically significant. Categorical variables are expressed as frequencies and percentages. Continuous variables are expressed as mean ± standard error (SE). The characteristics of the subjects in the low and high SVR groups were compared using the student’s *t*-test and Chi-squared test. The correlation between SO indicators and cardiometabolic risk factors was analyzed with the Pearson correlation. The comparison of cardiometabolic risk factors and dietary factors of the low and high SVR groups was performed using a general linear model adjusted for age, menopause status, total energy intake, alcohol consumption, smoking, physical activity, education level, and household members. Linear regression analysis was performed to analyze the linear relationship among the SVR, cardiometabolic risk factors, and dietary factors.

## 3. Results

Initially, 59 participants who satisfied the inclusion criteria and filled out the consent form were recruited. However, six were excluded from the analysis due to loss of dietary data, leaving 53 participants for the analysis. Table 1 shows the socio-demographic and health-related characteristics according to the SVR. We divided the 53 adult obese women into two groups according to their SVR values. There were 26 women in the low SVR group, with an average age of 53.4 ± 6.7 years, and 27 women in the high SVR group, with an average age of 51.7 ± 6.7 years. There were significant differences in total physical activity (MET) (*p* = 0.046) but not in other indicators. The low SVR group had lower total physical activity than the high SVR group (1632.31 ± 1280.10 METs vs. 2630.37 ± 2161.66 METs, *p* = 0.046). The group at a high risk of SO had a lower physical activity than the low risk group.

Table 2 shows the correlations between the SVR, SBR, SFR, SWR, and cardiometabolic risk factors. The SVR had inverse correlations with FBS, hs-CRP, total cholesterol, LDL-C, leptin, BMI, waist circumference, WHR, and total fat. Conversely, there was a positive correlation with physical activity. Analysis of the associations between the SVR, SBR, SFR, SWR, and cardiometabolic risk factors showed that the SVR had a closer association with cardiometabolic risk factors than the SBR, SFR, and SWR.

Table 3 shows the results of the comparative analysis of low and high SVR groups and cardiometabolic risk factors. After adjusting for the confounding factors, the low SVR group had significantly higher levels than the high SVR group among cardiometabolic risk factors: hs-CRP (low vs. high = 0.18 mg/dL vs. 0.07 mg/dL), total cholesterol (233.06 mg/dL vs. 197.87 mg/dL), LDL-C (142.38 mg/dL vs. 119.45 mg/dL), leptin (18.18 ng/mL vs. 11.98 ng/mL), BMI (28.19 kg/m^2^ vs. 27.04 kg/m^2^), visceral fat area (144.20 cm^2^ vs. 94.74 cm^2^), subcutaneous fat area (280.19 cm^2^ vs. 230.85 cm^2^), total fat percentage (43.59% vs. 39.79%), and fat mass (28.97 kg vs. 26.08 kg). Conversely, lean mass (low = 37.07 kg, high = 39.38 kg) was significantly higher in the high SVR group.

Table 4 shows the results of the average comparative analysis between the low and high SVR groups for the intake of each nutrient. After adjustment, the low SVR group had a higher intake of total fatty acids (low = 49.48 ± 3.32 g, high = 38.12 ± 3.25 g), monounsaturated fatty acids (MUFA) (low = 18.48 ± 1.58 g, high = 13.58 ± 1.55 g), and polyunsaturated fatty acids (PUFA) (low = 16.96 ± 1.15 g, high = 12.80 ± 1.13 g) than the high SVR group (*p* < 0.05), while the high SVR group had a higher intake of vitamin K (low = 162.39 ± 29.70 g, high = 258.09 ± 29.09 g) than the low SVR group (*p* < 0.05)**.** Overall, the obesity-related factors and inflammation index were higher and the blood lipid index was worse in the group closer to having SO. In addition, fatty acid intake was higher in that group.

Table 5 shows the association between the SVR value and cardiometabolic risk factors. In the unadjusted crude model, as the SVR value increased, the FBS (β = −0.278, *p* = 0.044), hs-CRP (β = −0.345, *p* = 0.011), total cholesterol (β = −0.379, *p* = 0.005), LDL-C (β = −0.360, *p* = 0.008), and leptin (β = −0.304, *p* = 0.027) levels decreased. Among the obesity-related factors, as the SVR value increased, the BMI (β = −0.322, *p* = 0.019), WC (β = −0.302, *p* = 0.028), WHR (β = −0.362, *p* = 0.008), and total fat (%) (β = −0.426, *p* = 0.001) values decreased. Conversely, among lifestyle factors, as total physical activity increased (β = 0.358, *p* = 0.008), the SVR value increased. After adjusting for confounders, FBS and leptin were no longer significant. In addition, the crude model showed significance in WC and WHR, which became non-significant after adjustment. The worse the blood lipid index, inflammatory index, and obesity-related index status, the more likely that an individual would have SO. 

Table 6 shows the association between dietary factors and the SVR value. After adjusting for confounders, among macronutrients, the SVR value decreased as the total fatty acid, saturated fatty acid (SFA), MUFA, and PUFA intake increased (β = −0.367, *p* = 0.012; β = −0.287, *p* = 0.044; β = −0.282, *p* = 0.048; β = −0.301, *p* = 0.035). Conversely, among water-soluble vitamins, the SVR value increased with the intake of vitamins B_6_ (β = 0.338, *p*= 0.012) and B_12_ (β = 0.281, *p*= 0.024). Therefore, as the intake of fatty acids increased, so did the risk of SO. Conversely, as the intake of vitamins B_6_ and B_12_ increased, the risk of SO decreased.

## 4. Discussion

This study showed that the SVR was negatively correlated with inflammatory indicators, blood lipids, and fatty acid intake. Conversely, it was positively correlated with physical activity and vitamins B_6_ and B_12_. Furthermore, we demonstrated that the SVR was closely related to cardiometabolic risk factors. Similar to other indicators of SO, the SVR does not have an exact cut-off. Previous studies have shown that individuals with the lowest SVR are very likely to have SO [13,18]. Studies on SO are conducted frequently; therefore, studies to determine the cut-off of SVR are needed.

The literature shows that there is a relationship between cardiometabolic risk factors and SO [19,20]. Research showed that the risk of cardiometabolic diseases was greater with SO than with sarcopenia or obesity alone [21]. In addition, another study revealed a relationship between body fat and one or more cardiometabolic risk factors; it was found to be greater in women than in men. The study also showed that visceral fat has a deeper relationship with cardiometabolic risk factors than fat in other areas [22]. Similarly, our study showed a correlation between the SVR, an SO index, and cardiometabolic risk factors. This may be because our study subjects were females with obesity, and the SVR contains an area of visceral fat. However, associations with insulin resistance and blood pressure among the cardiometabolic risk factors were not significant. Studies have shown that muscle fatty infiltration induces insulin resistance in individuals with obesity [23,24]. However, this study’s results differed from previous studies because we excluded people with fasting blood glucose levels above 126 mg/dL. Further, blood pressure association may have been non-significant because subjects with uncontrolled hypertension were excluded.

Similar to our findings, an earlier study showed that individuals closer to having SO have higher levels of hs-CRP, an inflammatory marker [20]. Research has shown that CRP differs depending on the muscle mass and fat localization in patients with SO. CRP concentration was inversely proportional to muscle mass (β = −0.629, *p* = 0.002) and proportional to total fat mass (β = 0.049, *p* < 0.001) [25]. In addition, higher visceral fat is strongly associated with inflammatory markers [26]. These previous studies found that low muscle mass and abdominal obesity affected hs-CRP. In this study, there was also an inverse correlation between the SVR and hs-CRP (β = −0.305, *p* = 0.043). Based on this, we observed that individuals with a low SVR had a higher inflammatory index because a low SVR value is associated with high visceral fat and low muscle mass.

A previous study showed a positive correlation between dietary intake of linoleic acid, a PUFA, and weight gain in women (OR: 1.56, 95% CI: 1.27, 1.90) [27]. Another study demonstrated that fish oil supplementation containing *n*-3 PUFAs increased muscle mass (treatment effect: 3.6%, 95% CI: 0.2%, 7.0%) and strength (4.0%, 95% CI: 0.8%, 7.3%) in people aged 60 years and older [28]. Thus, the same PUFA affects SO differently depending on the food from which it is derived. In our study, there was an inverse correlation between PUFA intake and the SVR (β = −0.301, *p* = 0.035). Here, we observed that higher PUFA intake was associated with a lower SVR. By analyzing the 3-day food records of the individuals, we concluded that subjects with a lower SVR mainly ate dishes fried in linoleic acid-rich soybean or corn oils. 

Research has shown that the intake of MUFA prevents abdominal obesity and lowers sarcopenia risk [29,30]. In contrast, in this study, there was an inverse correlation between MUFA and the SVR because the effect of MUFA on the body was affected by the content of dietary saturated fatty acids [31]. Therefore, it seems that the effect of MUFA on the body decreased because the intake of SFA was higher even if MUFA intake was high.

Earlier work showed that vitamins B_6_ and B_12_ lower homocysteine levels. Increased levels of homocysteine reduced muscle strength and walking speed [32]. Thus, vitamin B_6_ and B_12_ intake could prevent sarcopenia, which is also characterized by diminished muscle strength and walking speed. Vitamin B_6_ is a cofactor of glycogen kinase. This enzyme breaks down glycogen to produce energy, and low levels of this enzyme could affect body weight because of poor glycogen breakdown [33]. In addition, increased blood homocysteine levels, often seen in vitamin B_12_-deficient individuals with obesity, can affect energy metabolism and induce weight gain. Several studies have shown that individuals with obesity have a deficiency in vitamins B_6_ and B_12_ [34]. We consistently observed a positive correlation between the SVR and intake of vitamins B_6_ and B_12_, suggesting that a higher intake can lower the probability of developing SO.

Although it has been shown that protein intake could prevent SO [35,36], no association was observed between the SVR and protein intake in our study. This might be because there were only minor differences in energy and protein intake between individuals and the number of participants was small. According to a previous study, the abdominal fat area increased with increased animal protein intake [37]. In our study, the low SVR group consumed more animal protein than the high SVR group who consumed a more plant-based protein diet than the low SVR group (Table S1). However, the difference was not significant. Although no association between protein intake and SVR was observed in this study, our results suggest that the SVR may vary depending on the protein source.

Our study had limitations. Because this was a cross-sectional study, it is difficult to establish a causal relationship between SO, cardiometabolic risk factors, and dietary factors. Second, the number of subjects was small because it was an analysis of the baseline data of a previous study. The strength of our study is that it can predict SO more accurately by simultaneously considering skeletal muscle mass and visceral fat. In addition, it is novel in that it analyzes the correlations between the SVR and cardiometabolic health risk factors and between dietary factors. Unlike many other studies with only elderly participants, we included adults between the ages of 19 and 65. This study can support the value of the SVR as an indicator of SO.

## 5. Conclusions

The lower the SVR, which is more likely to occur in individuals with SO, the higher the blood lipids and levels of inflammatory markers. Among dietary factors, as the intake of vitamins B_6_ and B_12_ increases, the SVR increases, thus decreasing the chances of SO. Conversely, as the intake of fatty acids increases, individuals are at higher risk of SO.

## Figures and Tables

**Table 1:** Socio-demographic and health-related characteristics of the subjects in the low and high SVR groups.

Variables	Low SVR Group (*n* = 26)	High SVR Group (*n* = 27)	*p*-Value ^1,2^
Age (y) ^1^	53.4 ± 6.7	51.7 ± 6.7	0.365
Education level, *n* (%) ^2^			
≤Elementary school graduate	0 (0.00)	1 (3.70)	
Middle school graduate	2 (7.69)	3 (11.11)	
High school graduate	8 (30.77)	10 (37.04)	
≥University graduate	16 (61.54)	13 (48.15)	
Household members, *n* (%) ^2^			
First generation	4 (15.38)	6 (22.22)	
Second generation	20 (76.92)	21 (77.78)	
Third generation	2 (7.69)	0 (0.00)	
Menopause status, *n* (%) ^2^			0.449
Yes	18 (69.23)	16 (59.26)	
No	8 (30.77)	11 (40.74)	
Medical history, *n* (%) ^2^			0.928
Yes	8 (30.77)	8 (29.63)	
Hypertension	3 (11.54)	2 (7.41)	
Dyslipidemia	0 (0)	1 (3.70)	
Hypertension and dyslipidemia	2 (7.69)	3 (11.11)	
No	18 (69.23)	19 (70.37)	
Alcohol consumption, *n* (%) ^2^			0.947
Non-drinker	12 (46.15)	14 (51.85)	
Former drinker	2 (7.69)	2 (7.41)	
Current drinker	12 (46.15)	11 (40.74)	
Smoking, *n* (%) ^2^			0.530
Non-smoker	24 (92.31)	26 (96.3)	
Former smoker	-	-	
Current smoker	2 (7.69)	1 (3.70)	
Protein supplement intake, *n* (%) ^2^			0.322
Yes	0 (0.00)	1 (3.70)	
No	26 (100.00)	26 (96.30)	
Physical activity (GPAQ) ^1^			
Activity at work (METs)	36.92 ± 188.27	0.00 ± 0.00	0.327
Travel to and from places (METs)	1420.00 ± 1203.69	2263.70 ± 1836.46	0.053
Recreational activity (METs)	175.38 ± 346.52	366.67 ± 915.15	0.318
Total physical activity (METs)	1632.31 ± 1280.10	2630.37 ± 2161.66	0.046
Sedentary time (min)	471.92 ± 179.31	517.78 ± 192.20	0.374

Abbreviations: SVR, appendicular skeletal muscle mass-to-visceral fat area ratio; METs, metabolic equivalents of task. SVR: Low < 141.21 g/cm^2^, High ≥ 141.21 g/cm^2^. ^1^ Continuous variables are expressed as mean ± SD and the significant difference between continuous variables was measured with the student’s *t*-test (*p* < 0.05). ^2^ Categorical variables are expressed as *n* (%), and the significant difference between categorical variables was measured with the Chi-squared test (*p* < 0.05).

**Table 2:** Pearson correlations between SVR, SBR, SFR, SWR, and cardiometabolic risk factors.

	SVR		SBR		SFR		SWR	
Variables	r	P	r	P	r	P	r	P
Biochemical measurements								
FBS (mg/dL)	−0.278	0.044	−0.002	0.989	−0.187	0.180	−0.124	0.375
Insulin (uIU/mL)	−0.102	0.466	0.077	0.585	−0.112	0.425	−0.017	0.901
HOMA-IR	−0.149	0.287	0.061	0.663	−0.141	0.314	−0.044	0.756
hs-CRP (mg/L)	−0.345	0.012	−0.146	0.298	−0.402	0.003	−0.109	0.438
TG (mg/dL)	−0.187	0.180	−0.045	0.751	−0.150	0.284	−0.040	0.779
Total cholesterol (mg/dL)	−0.379	0.005	−0.196	0.159	−0.299	0.029	−0.184	0.188
HDL-C (mg/dL)	−0.038	0.790	−0.111	0.429	−0.081	0.562	−0.171	0.220
LDL-C (mg/dL)	−0.360	0.008	−0.172	0.218	−0.289	0.036	−0.133	0.343
Leptin (ng/mL)	−0.304	0.027	−0.381	0.005	−0.673	<0.0001	−0.389	0.004
Adiponectin (μg/mL)	0.063	0.655	−0.049	0.729	0.074	0.598	0.000	0.999
Blood pressure								
SBP (mmHg)	−0.104	0.460	0.075	0.596	0.106	0.451	−0.012	0.933
DBP (mmHg)	0.098	0.487	0.075	0.593	0.010	0.945	0.090	0.521
Obesity-related factors								
BMI (kg/m^2^)	−0.322	0.019	−0.277	0.045	−0.567	<0.0001	−0.177	0.205
Waist circumference (cm)	−0.302	0.028	−0.174	0.212	−0.448	0.001	−0.305	0.026
WHR	−0.362	0.008	−0.222	0.111	−0.148	0.291	−0.430	0.001
Total fat mass (kg)	−0.267	0.053	−0.224	0.107	−0.756	<0.0001	−0.158	0.259
Total fat (%)	−0.426	0.002	−0.634	<0.0001	−0.967	<0.0001	−0.564	<0.0001
Life-style factors								
Alcohol consumption	0.024	0.864	0.009	0.951	0.013	0.927	−0.010	0.945
Smoking Status	−0.118	0.400	−0.204	0.143	−0.070	0.620	−0.140	0.319
Physical activity (METs)	0.358	0.009	0.055	0.697	0.067	0.634	0.055	0.697

Abbreviations: SVR, appendicular skeletal muscle mass-to-visceral fat area ratio; SBR, appendicular skeletal muscle mass-to-BMI ratio; SFR, appendicular skeletal muscle mass-to-total fat mass ratio; SWR, appendicular skeletal muscle mass-to-waist ratio; FBS, fasting blood sugar; HOMA-IR, Homeostatic Model Assessment for Insulin Resistance; TG, triglyceride; HDL-C, high density lipoprotein cholesterol; LDL-C, low density lipoprotein cholesterol; SBP, systolic blood pressure; DBP, diastolic blood pressure; BMI, body mass index; WHR, waist–hip ratio; METs (min/week), Metabolic equivalents of task (min/week).

**Table 3:** Comparison of cardiometabolic risk factors between low and high SVR groups.

Variables	Low SVR Group (*n* = 26)	High SVR Group (*n* = 27)	*p*-Value ^1,2^
Biochemical measurements ^1^			
FBS (mg/dL)	97.74 ± 2.05	93.84 ± 2.01	0.199
Insulin (uIU/mL)	9.75 ± 0.76	7.94 ± 0.74	0.109
HOMA-IR	2.42 ± 0.21	1.85 ± 0.20	0.070
hs-CRP (mg/dL)	0.18 ± 0.02	0.07 ± 0.02	0.006
TG (mg/dL)	153.88 ± 16.07	129.71 ± 15.75	0.308
Total cholesterol (mg/dL)	233.06 ± 8.69	197.87 ± 8.52	0.008
HDL-C (mg/dL)	67.67 ± 2.58	64.02 ± 2.52	0.337
LDL-C (mg/dL)	142.38 ± 6.62	119.45 ± 6.49	0.022
Leptin (ng/mL)	18.18 ± 1.60	11.98 ± 1.56	0.011
Adiponectin (μg/mL)	8.25 ± 0.63	8.10 ± 0.62	0.875
Adiponectin/leptin ratio	0.66 ± 0.12	0.86 ± 0.12	0.268
Blood pressure ^1^			
SBP (mmHg)	122.70 ± 2.68	122.73 ± 2.63	0.993
DBP (mmHg)	79.09 ± 1.59	80.03 ± 1.56	0.688
Obesity-related factors ^1^			
Weight (kg)	69.18 ± 1.31	68.60 ± 1.28	0.761
Body mass index (kg/m^2^)	28.19 ± 0.36	27.04 ± 0.36	0.036
Waist circumference (cm)	91.81 ± 1.29	88.13 ± 1.27	0.057
Hip circumference (cm)	100.45 ± 0.94	99.70 ± 0.92	0.586
WHR	0.91 ± 0.01	0.88 ± 0.01	0.106
Visceral fat area (cm^2^)	144.20 ± 5.15	94.74 ± 5.04	<0.0001
Subcutaneous fat area (cm^2^)	280.19 ± 14.42	230.85 ± 14.13	0.024
Total fat (%)	43.59 ± 0.81	39.79 ± 0.79	0.003
Total fat mass (kg)	28.97 ± 0.90	26.08 ± 0.88	0.033
Total lean mass (kg)	37.07 ± 0.69	39.38 ± 0.67	0.026

Abbreviations: SBP, systolic blood pressure; SVR, appendicular skeletal muscle mass-to-visceral fat area ratio; DBP, diastolic blood pressure; WHR, waist–hip ratio; FBS, fasting blood sugar; HOMA-IR, Homeostatic Model Assessment for Insulin Resistance; TG, triglyceride; HDL-C, high-density lipoprotein cholesterol; LDL-C, low-density lipoprotein cholesterol. SVR: Low < 141.21 g/cm^2^, High ≥ 141.21 g/cm^2^. ^1^ Variables are expressed as mean ± SE and obtained using general linear model at *p* < 0.05. ^2^ Adjusted: age, menopause status, alcohol consumption, smoking, physical activity, education level, household member.

**Table 4:** Comparison of nutrient intake between low and high SVR groups.

Variables	Low SVR Group (*n* = 26)	High SVR Group (*n* = 27)	*p*-Value ^1,2^
Energy (Kcal/day) ^1^	2007.68 ± 112.23	2250.47 ± 109.97	0.144
Macronutrients ^1^			
Carbohydrates (g/day)	268.73 ± 8.26	285.25 ± 8.09	0.180
Protein (g/day)	86.74 ± 4.23	84.14 ± 4.14	0.677
Fat (g/day)	69.55 ± 2.78	66.23 ± 2.72	0.420
Fiber (g/day)	25.48 ± 1.49	27.05 ± 1.46	0.479
C:P:F ratio (%)	51:17:13	54:16:12	
Cholesterol (mg/day)	446.42 ± 35.51	364.31 ± 34.78	0.123
Total FA (g/day)	49.48 ± 3.32	38.12 ± 3.25	0.024
SFA (g/day)	13.79 ± 1.29	11.86 ± 1.27	0.315
MUFA (g/day)	18.48 ± 1.58	13.58 ± 1.55	0.040
PUFA (g/day)	16.96 ± 1.15	12.80 ± 1.13	0.018
MUFA/SFA ratio	1.36 ± 0.06	1.20 ± 0.06	0.065
ω-3 FA (g/day)	0.75 ± 0.16	0.55 ± 0.16	0.395
ω-6 FA (g/day)	4.52 ± 0.93	3.13 ± 0.92	0.318
ω-3 FA/ω-6 FA ratio	0.20 ± 0.06	0.22 ± 0.06	0.830
Fat-soluble Vitamins ^1^			
Vitamin A (μg RAE/day)	469.56 ± 46.69	593.00 ± 45.73	0.079
Vitamin D (μg/day)	5.41 ± 2.29	6.49 ± 2.25	0.749
Vitamin E (mg/day)	23.96 ± 1.35	22.41 ± 1.33	0.439
Vitamin K (μg/day)	162.39 ± 29.70	258.09 ± 29.09	0.033
Water-soluble Vitamins ^1^			
Thiamin (mg/day)	1.99 ± 0.10	2.03 ± 0.10	0.792
Riboflavin (mg/day)	1.65 ± 0.06	1.65 ± 0.06	0.959
Niacin (mg/day)	16.08 ± 1.22	16.22 ± 1.19	0.936
Pantothenic acid (mg/day)	4.78 ± 0.18	4.59 ± 0.18	0.494
Vitamin B_6_ (mg/day)	1.81 ± 0.40	3.00 ± 0.39	0.050
Vitamin B_12_ (μg/day)	10.58 ± 2.20	14.55 ± 2.15	0.226
Vitamin C (mg/day)	111.80 ± 14.39	125.36 ± 14.09	0.524
Folate (μg/day)	534.11 ± 32.70	546.52 ± 32.03	0.797
Minerals (mg/day) ^1^			
Ca	502.67 ± 33.13	591.64 ± 32.45	0.074
P	1294.03 ± 59.35	1259.32 ± 58.13	0.692
Na	4504.55 ± 276.43	3942.64 ± 270.75	0.173
K	2880.69 ± 160.26	3238.61 ± 156.96	0.135
Mg	132.26 ± 10.54	129.51 ± 10.32	0.860
Fe	20.16 ± 1.59	19.28 ± 1.55	0.706

Abbreviations: C:P:F ratio, ratio of carbohydrate to protein to fat; Total FA, total fatty acid; SFA, saturated fatty acid; SVR, appendicular skeletal muscle mass-to-visceral fat area ratio; MUFA, monounsaturated fatty acid; PUFA, polyunsaturated fatty acid; ω-3 FA, omega-3 fatty acid; ω-6 FA, omega-6 fatty acid; Ca, calcium; P, phosphorus; Na, sodium; K, potassium; Mg, magnesium; Fe, Iron. SVR: Low < 141.21 g/cm2, High ≥ 141.21 g/cm^2^. ^1^ Variables are expressed as mean ± SE and obtained using general linear model at *p* < 0.05. ^2^ Adjusted: age, menopause status, total energy intake, physical activity, education level, household member.

**Table 5:** The linear associations between SVR values and cardiometabolic risk factors.

	SVR Value							
	Crude				Multivariate ^a^			
	β	B	SE	*p* ^1^	β	B	SE	*p* ^1^
Biochemical measurements ^1^								
FBS (mg/dL)	−0.278	−0.056	0.027	0.044	−0.174	−0.035	0.035	0.316
Insulin (uIU/mL)	−0.102	−0.009	0.012	0.466	−0.033	−0.003	0.013	0.824
HOMA-IR	−0.149	−0.004	0.003	0.287	−0.068	−0.002	0.004	0.655
hs-CRP (mg/dL)	−0.345	−0.001	0.000	0.011	−0.305	−0.001	0.000	0.043
TG (mg/dL)	−0.187	−0.344	0.253	0.180	−0.071	−0.131	0.286	0.648
Total cholesterol (mg/dL)	−0.379	−0.355	0.121	0.005	−0.369	−0.346	0.145	0.022
HDL-C (mg/dL)	−0.038	−0.010	0.039	0.790	−0.102	−0.028	0.045	0.530
LDL-C (mg/dL)	−0.360	−0.248	0.090	0.008	−0.326	−0.225	0.111	0.049
Leptin (ng/mL)	−0.304	−0.052	0.023	0.027	−0.252	−0.043	0.025	0.097
Adiponectin (μg/mL)	0.063	0.004	0.010	0.655	−0.133	−0.009	0.011	0.401
Blood pressure ^1^								
SBP (mmHg)	−0.104	−0.028	0.037	0.460	−0.209	−0.056	0.046	0.236
DBP (mmHg)	0.098	0.016	0.023	0.487	−0.045	−0.008	0.028	0.788
Obesity-related factors ^1^								
BMI (kg/m^2^)	−0.322	−0.013	0.005	0.019	−0.362	−0.014	0.006	0.020
Waist circumference (cm)	−0.302	−0.040	0.017	0.028	−0.058	−0.008	0.013	0.563
WHR	−0.362	0.000	0.000	0.008	−0.184	0.000	0.000	0.228
Total fat mass (kg)	−0.267	−0.025	0.013	0.053	−0.074	−0.007	0.008	0.368
Total fat (%)	−0.426	−0.036	0.011	0.001	−0.330	−0.028	0.011	0.011
Lifestyle factors ^1^								
Alcohol consumption	0.024	0.001	0.004	0.864	−0.124	−0.004	0.005	0.434
Smoking	−0.118	−0.001	0.001	0.400	−0.135	−0.001	0.002	0.434
Physical activity (METs)	0.358	0.009	0.003	0.008	0.390	0.010	0.004	0.011

Abbreviations: FBS, fasting blood sugar; HOMA-IR, Homeostatic Model Assessment for Insulin Resistance; SVR, appendicular skeletal muscle mass-to-visceral fat area ratio; TG, triglyceride; HDL-C, high density lipoprotein cholesterol; LDL-C, low density lipoprotein cholesterol; SBP, systolic blood pressure; DBP, diastolic blood pressure; BMI, body mass index; WHR, waist–hip ratio; METs (min/week), Metabolic equivalents of task (min/week). ^1^ Values are expressed as standardized coefficients (β), unstandardized coefficients (B), SE and obtained by linear regression analysis at *p* < 0.05. ^a^ Adjusted: age, menopause status, BMI, alcohol consumption, smoking, physical activity, education level, household members, energy.

**Table 6:** The linear associations between dietary factors and SVR values.

	SVR Value							
	Crude				Multivariate ^a^			
	β	B	SE	*p* ^1^	β	B	SE	*p* ^1^
Energy (Kcal) ^1^	0.012	0.011	0.012	0.387	0.166	0.015	0.012	0.226
Macronutrients ^1^								
Carbohydrates (g)	0.214	0.145	0.092	0.124	0.434	0.293	0.157	0.068
Protein (g)	0.043	0.081	0.264	0.760	0.103	0.195	0.318	0.544
Fat (g)	0.094	0.159	0.238	0.505	−0.161	−0.274	0.481	0.572
Cholesterol (mg)	−0.011	−0.003	0.036	0.937	−0.098	−0.025	0.037	0.494
Total FA (g)	−0.208	−0.529	0.348	0.135	−0.367	−0.933	0.356	0.012
SFA (g)	−0.144	−1.030	0.989	0.303	−0.287	−2.047	0.985	0.044
MUFA (g)	−0.205	−1.152	0.770	0.141	−0.282	−1.581	0.779	0.048
PUFA (g)	−0.166	−1.250	1.039	0.235	−0.301	−2.268	1.045	0.035
ω−3 FA (g)	−0.087	−5.358	8.548	0.534	−0.016	−1.003	8.502	0.907
ω−6 FA (g)	−0.105	−1.120	1.487	0.455	−0.076	−0.814	1.421	0.569
Fat-soluble vitamins ^1^								
Vitamin A (μg RAE)	0.217	0.038	0.024	0.119	0.207	0.036	0.028	0.198
Vitamin D (μg)	0.074	0.338	0.634	0.596	0.058	0.265	0.585	0.653
Vitamin E (mg)	0.033	0.187	0.784	0.813	−0.047	−0.264	1.000	0.793
Vitamin K (μg)	0.193	0.057	0.041	0.166	0.228	0.067	0.042	0.114
Water-soluble vitamins ^1^								
Thiamin (mg)	0.102	6.459	8.804	0.467	−0.129	−8.163	12.955	0.532
Riboflavin (mg)	0.165	15.224	12.740	0.238	0.134	12.318	22.243	0.583
Niacin (mg)	0.060	0.475	1.099	0.668	0.090	0.706	1.097	0.523
Pantothenic acid (mg)	0.134	5.104	5.297	0.340	−0.069	−2.644	7.408	0.723
Vitamin B_6_ (mg)	0.169	3.943	3.223	0.227	0.338	7.903	3.000	0.012
Vitamin B_12_ (μg)	0.253	1.197	0.642	0.068	0.281	1.329	0.570	0.024
Vitamin C (mg)	0.139	0.093	0.093	0.320	0.088	0.059	0.093	0.532
Folate (μg)	0.027	0.007	0.035	0.846	0.030	0.008	0.041	0.854
Minerals ^1^								
Ca (mg)	0.170	0.039	0.032	0.223	0.260	0.060	0.038	0.124
P (mg)	−0.053	−0.007	0.018	0.707	0.040	0.005	0.023	0.820
Na (mg)	0.054	0.002	0.005	0.698	0.010	0.000	0.005	0.947
K (mg)	0.149	0.007	0.007	0.287	0.320	0.016	0.008	0.055
Mg (mg)	−0.047	−0.039	0.117	0.737	0.057	0.047	0.127	0.713
Fe (mg)	0.098	0.528	0.750	0.485	0.068	0.366	0.847	0.668
Eating behavior ^1^								
Restrained Eating	0.037	2.064	7.876	0.794	0.080	4.516	7.142	0.530
Emotional Eating	0.012	0.769	8.903	0.932	0.017	1.076	8.285	0.897
External Eating	0.081	6.967	11.968	0.563	0.085	7.291	11.530	0.530

Abbreviations: SVR, appendicular skeletal muscle mass-to-visceral fat area ratio; Total FA, total fatty acid; SFA, saturated fatty acid; MUFA, monounsaturated fatty acid; PUFA, polyunsaturated fatty acid; ω-3 FA, omega-3 fatty acid; ω-6 FA, omega-6 fatty acid; Ca, calcium; P, phosphorus; Na, sodium; K, potassium; Mg, magnesium; Fe, Iron. ^1^ Values are expressed as standardized coefficients (β), unstandardized coefficients (B), SE and obtained by linear regression analysis at *p* < 0.05. ^a^ Adjusted: age, menopause status, total energy, physical activity, BMI, education level, household member.

## Data Availability

The data are not publicly available due to [ethical restrictions].

## Supplementary Materials

The following are available online at https://www.mdpi.com/article/10.3390/nu15071574/s1, Table S1: Comparison of average daily food intake (g) between low and high SVR groups.
