# Supplementary material for: Association between Skeletal Muscle Mass-to-Visceral Fat Ratio and Dietary and Cardiometabolic Health Risk Factors among Korean Women with Obesity
Source: Nutrients. 2023 Mar 24;15(7):1574. doi: 10.3390/nu15071574 (PMC10097179; doi:10.3390/nu15071574)
Supplement: Supplementary file 1 [file nutrients-15-01574-s001.zip › nutrients-2262711-supplementary.pdf]

Table S1. Comparison of average daily food intake (g) between low and high SVR

Variables	Low SVR Group (n = 26)			High SVR Group (n = 27)			p-Value [1,2]
Grains (g) [1]	289.39	±	15.96	297.86	±	15.63	0.720
Potatoes (g) [1]	59.50	±	13.63	53.50	±	13.35	0.766
Sugars (g) [1]	18.11	±	4.07	16.91	±	3.99	0.841
Legumes (g) [1]	46.49	±	12.66	43.81	±	12.40	0.886
Nuts and seeds (g) [1]	9.06	±	6.46	10.87	±	6.32	0.850
Vegetables (g) [1]	293.47	±	28.42	339.67	±	27.83	0.274
Mushrooms (g) [1]	9.60	±	2.88	5.12	±	2.82	0.294
Fruits (g) [1]	118.18	±	32.53	189.81	±	31.86	0.141
Meats (g) [1]	142.65	±	14.43	105.26	±	14.14	0.085
Eggs (g) [1]	59.14	±	7.30	36.37	±	7.15	0.039
Fishes and shellfishes (g) [1]	85.50	±	24.21	93.48	±	23.71	0.823
Seaweeds (g) [1]	4.67	±	1.64	3.75	±	1.61	0.703
Milk and dairy products (g) [1]	54.23	±	14.58	88.54	±	14.29	0.116
Oils and fats (g) [1]	16.10	±	1.32	13.22	±	1.29	0.144
Beverages (g) [1]	47.17	±	20.60	45.03	±	20.18	0.944
Seasonings (g) [1]	34.71	±	3.67	37.04	±	3.60	0.668
Coffee (g) [1]	195.27	±	43.42	262.64	±	42.53	0.296
Tea (g) [1]	12.08	±	17.59	24.42	±	17.23	0.635
Alcohol (g) [1]	76.07	±	26.69	35.81	±	26.14	0.310

SVR: Low < 141.21 g/cm^2, High ≥ 141.21 g/cm^2

[1] Variables are expressed as mean ± SE and obtained using general linear model at $p < 0.05$.

[2] Adjusted: age, menopause status, total energy, physical activity, education level, household member
